# Volume Fraction Determination of Binary Liquid Mixtures by Measurement of the Equalization Wavelength

**DOI:** 10.3390/s100807082

**Published:** 2010-07-27

**Authors:** Ivan Martincek, Dusan Pudis, Daniel Kacik, Kay Schuster

**Affiliations:** 1 Department of Physics, University of Žilina, Univerzitná 1, SK- 01026, Žilina, Slovakia; E-Mails: ivmar@fel.uniza.sk (I.M.); kacik@fel.uniza.sk (D.K.); 2 Institute of Photonic Technology Jena, D-07745, Jena, Germany; E-Mail: kay.schuster@ipht-jena.de

**Keywords:** intermodal interference, equalization wavelength, mixture volume fraction

## Abstract

A method for determination of the volume fraction in binary liquid mixtures by measurement of the equalization wavelength of intermodal interference of modes LP_01_ and LP_11_ in a liquid core optical fiber is presented in this paper. This method was studied using a liquid core optical fiber with fused silica cladding and a core made up of a binary silicon oil/chloroform liquid mixture with different volume fractions of chloroform. The interference technique used allows us to determine the chloroform volume fraction in the binary mixture with accuracy better than 0.1%. One of the most attractive advantages of presented method is very small volume of investigated mixture needed, as only a few hundred picoliters are necessary for reliable results.

## 1. Introduction

The composition of binary liquid mixtures is an important parameter for chemical and pharmaceutical applications [1,2], biochemical engineering [3], optical applications [4,5] *etc.* The refractive index is a function of the composition of binary liquid mixtures so their composition can be determined by refractive indices measurements [6].

Interferometric methods are optical methods which can determine the refractive index changes with mixtures’ compositions [7]. One of these interferometric methods is intermodal interference in optical fibers, which is often employed in optical fiber sensor design and can be used for characterization of optical fibers [8–10].

An important parameter of intermodal interference is the equalization wavelength *λ_e_* of two interfering modes [10]. For weakly-guiding optical fibers the value of the equalization wavelength depends on the core refractive index *n_co_* of the optical fiber, on the refractive index of the cladding *n_cl_* and on the core radius *ρ* [10]. A change of one of these three parameters can be readily detected by equalization wavelength *λ_e_* measurements.

In this paper we propose the use of the equalization wavelength measurement *λ_e_* of intermodal interference of interfering modes LP_01_ and LP_11_ in a liquid core optical fiber (LCOF), consisting of a binary liquid mixture, as a tool for determination of the liquid mixture’s composition. The different compositions of the binary liquid mixture results in ore refractive index changes, and hence it affects the value of the equalization wavelength. Then, by measurement of *λ_e_* in the LCOF the composition of liquid mixtures can be determined. This method can be then used favorably for investigation of small mixture volumes on the order of a few hundreds of picoliters.

## 2. Approach

For a weakly-guiding optical waveguide (*n_co_* ≈ *n_cl_*) with cylindrical symmetry and step refractive index profile of core and cladding the measured interference signal for intermodal interference of LP_01_ and LP_11_ modes can be expressed as a function of wavelength *λ*:

(1) $$\begin{array}{cc} P_{\text{int}}(\lambda ) & =\pi n_{\mathrm{co}}(\lambda )\rho^{2}\sqrt{\frac{{ɛ}_{0}}{\mu_{0}}}a_{1}(\lambda )a_{2}(\lambda )\;\text{cos}[(\beta_{1}\;(\lambda )-\beta_{2}\;(\lambda ))z].\\ & \cdot \int_{R}J_{0}\left(U_{1}\;(\lambda )\;R\right)J_{1}\;\left(U_{2}\;(\lambda )\;R\right)\;\mathrm{RdR}\int_{\varphi }\text{cos}\varphi d\varphi \end{array}$$

where *n_co_* is the core refractive index, *ɛ*_0_ and *μ*_0_ are the permittivity and permeability of free space, respectively, *a*_1_, *a*_2_ are the amplitude of LP_01_ and LP_11_ modes, *β*_1_ and *β*_2_ are the phase constants of LP_01_ and LP_11_ modes, *J*_0_, *J*_1_ are the Bessel functions of the first kind zero and first-order, *R* = *r*/*ρ*, where *ρ* is the core radius of optical fiber, *r* and *φ* are the polar coordinates, *U*_1_, *U*_2_ are the normalized propagation constants in the transverse direction in the core of LP_01_ and LP_11_ modes given by $U_{j}^{2}=\rho^{2}\left(k^{2}n_{\mathrm{co}}^{2}-\beta_{j}^{2}\right)$, where *k* = 2π/*λ* and *λ* is the wavelength in free space.

Phase constant of *j*th modes *β_j_* can be expressed as:

(2) $$\beta_{j}=k\sqrt{n_{\mathrm{co}}^{2}-\frac{U_{j}^{2}(V)}{V^{2}}(n_{\mathrm{co}}^{2}-n_{\mathrm{cl}}^{2})}$$

where *V* is normalized frequency defined by equation:

(3) $$V=k\rho \sqrt{n_{\mathrm{co}}^{2}-n_{\mathrm{cl}}^{2}}$$

If we suppose the weak dependence of *n_co_*, *n_cl_*, *a*_1_, *a*_2_, *J*_0_(*U*_1_ *R*) and *J*_1_(*U*_2_ *R*) on wavelength in Equation (1), then the interference signal *P*_int_ is a harmonic function:

(4) $$P_{\text{int}}(\lambda )\approx \text{cos}[(\beta_{1}(\lambda )-\beta_{2}(\lambda ))z]$$

Equation (4) indicates that the spectral dependence of interference signal of the two modes mainly depends on the length of fiber *z* and the difference of phase constants *β*_1_ and *β*_2_ of interfering modes. The difference of the phase constants *β*_1_ and *β*_2_ shows an extreme in wavelength dependence, which corresponds to the equalization wavelength [10]. According to Equations (2) and (3), the difference of phase constants of two modes depends on the core radius *ρ* and the respective refractive indices *n_co_*, *n_cl_*. This fact indicates, that for a step index optical fiber with constant core radius *ρ* and constant cladding refractive index *n_cl_*, the value of the equalization wavelength *λ_e_* is only a function of the refractive index of the core *n_co_*.

The refractive index of a binary liquid mixture depends on its individual component composition. If a binary liquid mixture constitutes the core of a step index optical fiber with constant radius *ρ* and with the constant cladding refractive index *n_cl_*, the value of the equalization wavelength *λ_e_* only depends on the refractive index of the binary mixture and consequently on its composition. The measurement of equalization wavelength *λ_e_* of interfering modes can thus determine the composition of binary liquid mixtures.

## 3. Experimental

**Figure 1.**
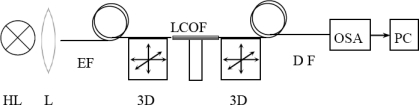
Experimental configuration for local spectral analysis of LCOF—liquid core optical fiber, HL—halogen lamp, L—lens, EF—excitation optical fiber, DF—detection optical fiber, 3D—nanopositioning stage, OSA—optical spectral analyzer, PC—personal computer.

The experimental setup for investigation of the intermodal interference of modes LP_01_ and LP_11_ in the liquid-core optical fiber is shown in Figure 1. The light from the halogen lamp is focused to the exciting optical fiber with the core diameter of 10 μm (SMF 28) and fixed on a positioning stage with the possibility of 3D axis motion.

**Figure 2.**
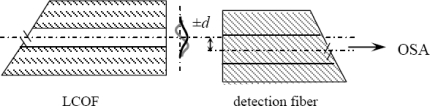
Experimental arrangement for detection of intermodal interference of the modes LP_01_ and LP_11_ in the LCOF.

Such an arrangement allows for optimal excitation of the LP_01_ and LP_11_ modes in the LCOF. The optical field of the LCOF is detected by detecting optical fiber with a core diameter of 10 μm. the signal from the detecting fiber is analyzed in an Ocean Optics USB2000 spectrometer operating in the 350–1,000 nm wavelength range and with a spectral resolution of 1.5 nm. The core of the detecting fiber was off-centered by a distance *d* = 2.8 μm from the LCOF axis (Figure 2) in order to detect the intermodal interference of modes LP_01_ and LP_11_.

The refractive index of the core and cladding of the LCOF is dependent on the temperature and hence temperature affects the equalization wavelength. Thus the measurement of equalization wavelength in the LCOF is necessary to thermally stabilize. In our experiment we fixed the temperature at 22.5 °C.

In the experiment we demonstrate the determination of binary liquid mixture composition by the measurement of the equalization wavelength of intermodal interference of modes LP_01_ and LP_11_ in a LCOF with lengths in the range of *z* = 16.0–60.1 mm. The investigated LCOF consists of a fused silica cladding and a binary liquid mixture core made up of silicon oil/chloroform with radius *ρ* = 2.75 μm. The mixture volume was 380 to 1,480 picoliters for the used lengths of LCOF. Reference Lukooil MF silicon oil (Lachema Brno, Czech Republic) was used in our experiment. The equalization wavelength *λ_e_* of this oil at the temperature 22.5 °C is in the spectral range of the spectrometer used.

**Figure 3.**
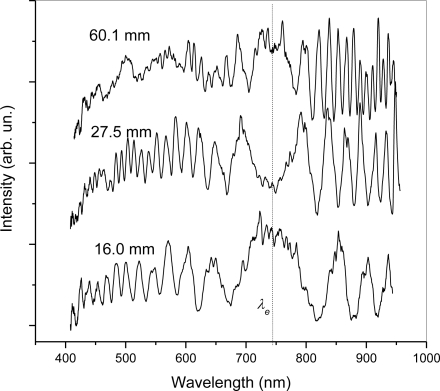
Interference signal for LCOF filled with pure silicon oil measured for different LCOF lengths.

The interference signal of modes LP_01_ and LP_11_ with the highlighted equalization wavelength *λ_e_* of the investigated LCOF filled with the silicon oil is shown in Figure 3. The influence of LCOF lengths of 16.0, 27.5 and 60.1 mm is demonstrated. These dependencies document the stable equalization wavelength *λ_e_*. Only the density of oscillations increases with the increasing of the LCOF length, which corresponds with the theoretical assumption.

**Figure 4.**
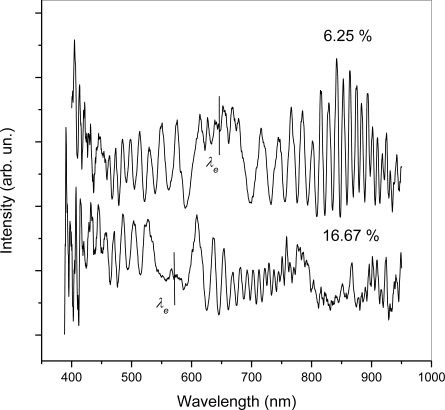
Shift of equalization wavelength shown on the measured spectra for the different volume fractions of chloroform in silicon oil.

Figure 4 shows the interference signal in LCOF with lengths 25.70 mm and 27.45 mm for two silicon oil/chloroform liquid mixtures with volume fractions of 6.25% and 16.67%, respectively. As the chloroform concentration in the mixture increases the equalization wavelength *λ_e_* shows an evident blue shift caused by a decrease of the refractive index of the binary mixture.

**Figure 5.**
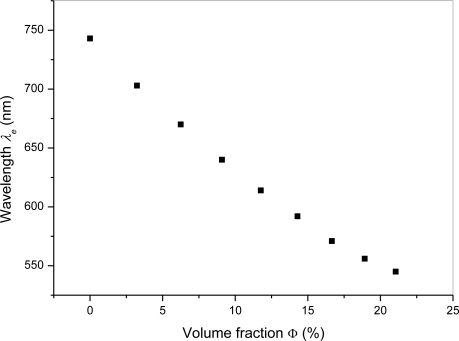
Measured dependence of equalization wavelength *λ_e_* on the volume fraction in the binary mixture.

The interference of the different liquid mixtures was investigated with volume fractions ranging from 0 % up to 21.05% of chloroform in the binary mixture. The equalization wavelength dependence *λ_e_* for relevant mixtures has been summarized in Figure 5.

**Figure 6.**
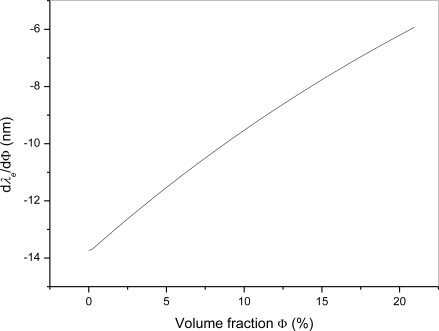
Calculated sensitivity of equalization wavelength *λ_e_* on the volume fraction in the investigated binary liquid mixture.

As shown in the dependence of the equalization wavelength on volume fraction of chloroform in the binary mixture the equalization wavelength exhibits a blue shift from 743 nm to 545 nm as the volume fraction increases from 0% to 21.05%. The calculated dependence of d*λ_e_*/dΦ from the acquired data with chloroform volume fraction in the binary mixture is presented in Figure 6. In the investigated range d*λ_e_*/dΦ changes from −13.7 nm to −5.9 nm. Using the interference technique we are able to measure the equalization wavelength with ±0.5 nm resolution, which allows us to determine the chloroform volume fraction in the binary mixture with an accuracy better than 0.1% in the range up to 7%. For higher concentrations the accuracy of this technique decreases and is lower than 0.2% in the whole investigated range.

## 4. Conclusions

In this paper we describe a method for determination of the composition of binary liquid mixtures by measurement of the equalization wavelength *λ_e_* of intermodal interference of modes LP_01_ and LP_11_ in a LCOF. This method was documented on a LCOF whose cladding consists of fused silica and the core consists of a binary liquid mixture of silicon oil/chloroform at different volume fraction of chloroform.

We favor this method for concentration determination in optically transparent mixtures for the investigated wavelength range because the investigated mixture constitutes the core of the LCOF. Also the refractive index of the mixture has to be higher than the refractive index of the cladding and the equalization wavelength *λ_e_* should be well detected in the investigated wavelength range. In the case that the condition of a lower cladding refractive index is not satisfied different materials e.g., other optical glasses and plastic could be used as a cladding.

One of the most attractive advantages of presented method is the very small volume of the investigated mixture needed. Only a few hundreds of picoliters are necessary for reliable results. This interference method for concentration determination in binary mixtures of picoliter volumes might see use in design of microfluidic devices such as “Labs-on-a-chip”.
